# Speed and Accuracy of a Point of Care Web-Based Knowledge Resource for Clinicians: A Controlled Crossover Trial

**DOI:** 10.2196/ijmr.2811

**Published:** 2014-02-21

**Authors:** David A Cook, Felicity Enders, Jane A Linderbaum, Dale Zwart, Farrell J Lloyd

**Affiliations:** ^1^Division of General Internal MedicineMayo Clinic College of MedicineRochester, MNUnited States; ^2^Knowledge Delivery CenterMayo Clinic College of MedicineRochester, MNUnited States; ^3^Department of Health Sciences ResearchMayo Clinic College of MedicineRochester, MNUnited States; ^4^Division of Cardiovascular DiseasesMayo Clinic College of MedicineRochester, MNUnited States; ^5^Alpinspire, LLCLittleton, COUnited States

**Keywords:** medical education, Web-based learning, educational technology, clinical decision support, health information technology

## Abstract

**Background:**

Effective knowledge translation at the point of care requires that clinicians quickly find correct answers to clinical questions, and that they have appropriate confidence in their answers. Web-based knowledge resources can facilitate this process.

**Objective:**

The objective of our study was to evaluate a novel Web-based knowledge resource in comparison with other available Web-based resources, using outcomes of accuracy, time, and confidence.

**Methods:**

We conducted a controlled, crossover trial involving 59 practicing clinicians. Each participant answered questions related to two clinical scenarios. For one scenario, participants used a locally developed Web-based resource, and for the second scenario, they used other self-selected Web-based resources. The local knowledge resource (“AskMayoExpert”) was designed to provide very concise evidence-based answers to commonly asked clinical questions. Outcomes included time to a correct response with at least 80% confidence (primary outcome), accuracy, time, and confidence.

**Results:**

Answers were more often accurate when using the local resource than when using other Web-based resources, with odds ratio 6.2 (95% CI 2.6-14.5; *P*<.001) when averaged across scenarios. Time to find an answer was faster, and confidence in that answer was consistently higher, for the local resource (*P*<.001). Overconfidence was also less frequent with the local resource. In a time-to-event analysis, the chance of responding correctly with at least 80% confidence was 2.5 times greater when using the local resource than with other resources (95% CI 1.6-3.8; *P*<.001).

**Conclusions:**

Clinicians using a Web-based knowledge resource designed to provide quick, concise answers at the point of care found answers with greater accuracy and confidence than when using other self-selected Web-based resources. Further study to improve the design and implementation of knowledge resources may improve point of care learning.

## Introduction

### Point of Care Questions

Ongoing advances in clinical medicine create new opportunities for patient-centered, high-value, personalized care, but the realization of this potential will require new models for translating evidence into practice. Clinicians frequently identify knowledge gaps while seeing patients [1,2], but many such point of care questions remain unanswered because busy clinicians cannot find answers in a timely fashion [3-5]. Increased speed and ease in finding accurate answers would improve practice efficiency and productivity; and over time might prompt clinicians to seek point of care information support as a routine part of their daily practice. In addition to speed and accuracy, effective knowledge translation requires that clinicians be appropriately confident in the answers they find—both overconfidence and lack of confidence will lead to suboptimal care [6].

Web-based knowledge resources can facilitate the translation of evidence into point of care practice [7], but current resources do not optimally address the potentially conflicting requirements of concise, complete, timely, balanced, and practical information [8-11]. To address these needs, we have developed a knowledge resource—“AskMayoExpert”—designed to provide very concise evidence-based answers to clinical questions (Textbox 1) [12]. The "frequently asked questions" (FAQ) feature of this multifaceted resource offers highly synthesized synopses of evidence [13] to satisfy focused point of care information needs. A comprehensive description and initial evaluation of AskMayoExpert has been published separately [12]; the present paper describes a study evaluating AskMayoExpert's FAQ feature.

### Purpose of the Present Study

The purpose of the present study was to evaluate this new knowledge resource in comparison with other available Web-based resources (such as, but not limited to or specifically targeting, UpToDate, MD Consult, PubMed, and Google). We hypothesized that the local resource would facilitate faster and equally accurate answers to clinical questions.

**Development and features of the AskMayoExpert Web-based knowledge resource.**

The AskMayoExpert Web-based knowledge resource [12] provides highly synthesized synopses of evidence to support rapid, accurate point of care decision making, and to facilitate the development of “gist” learning for long-term retention [14]. Each evidence synopsis is written as an answer to a common clinical FAQ, and is targeted to the needs and background understanding of a nonspecialist in that topic. All content is reviewed, revised, and approved by a content board of subspecialist experts and a senior physician editor, and is reviewed at least annually. Institutional leaders have endorsed this information as a quality standard for the entire institution.

Topics and FAQs have been added gradually, with priority determined by frequency, implications of mismanagement, and novelty of information (common, serious, and new/controversial topics receive top priority). At the time of this study AskMayoExpert contained 2478 FAQs on 490 disease-oriented topics.

Additional features (not relevant to the present study) include a directory of local topic experts, care process models (algorithms describing institution-approved ideal care pathways), clinical notifications of urgent test results, and patient education information. AskMayoExpert is available on the institution Intranet.

## Methods

### Overview and Setting

We conducted a controlled crossover trial in which clinicians answered one case-based question using a locally developed resource designed to provide concise answers, and another question using other Web-based resources of their choosing. The study took place at campuses of an academic medical center in Rochester, Minnesota; Jacksonville, Florida; and Scottsdale, Arizona, and an affiliated primary care clinic in Mankato, Minnesota, during March and April 2009. All staff at all sites have institution-sponsored access to several commercial knowledge resources including UpToDate, MD Consult, and Micromedex, in addition to publicly available Web-based resources. The Mayo Clinic Institutional Review Board approved the study protocol.

### Independent Variable

We created paper booklets containing two brief clinical scenarios (one scenario for each knowledge resource condition; Textbox 2), each with one key question about management. Scenario A focused on a common problem that is often managed without consideration of current evidence (atrial fibrillation—indications for stroke prevention anticoagulation), while Scenario B focused on an infrequently diagnosed condition for which management would be unfamiliar (apical ballooning syndrome—timing of follow-up). We created two versions of the booklets, one with Scenario A coming first (booklet A), and the other with Scenario B coming first (booklet B). Each booklet instructed participants to use AskMayoExpert to answer the first question, and to use any other Web-based resource to answer the second question (crossover design). Rather than selecting a specific "other" resource, we allowed participants to make this choice so that they could use a resource they felt was likely to give them an answer and with which they were comfortable.

**Outcome measures—scenarios and questions.**

Scenario A.

Please answer the following question using [assigned format].

A 56-year-old male was readmitted to the hospital with his second episode of atrial fibrillation and a rapid ventricular response in the last 2 months. He has severe sleep apnea and he uses CPAP^a^ at home. There is no prior history of stroke, coronary artery disease, diabetes, or hypertension. He is a one-pack-per-day smoker, but is trying to quit (20 pack-years). He began taking diltiazem, metoprolol and aspirin after his first episode one month ago. His initial blood pressure is 110/70 and his heart rate is 110.

Record start time—

*Mark only one best answer*—The moderate or high-risk indication for stroke prevention using Coumadin and not aspirin is which of the following? CPAP: continuous positive airway pressure; ECG: electrocardiogram; CCU: coronary care unit; Echo: echocardiogram; EF: ejection fraction.

1. Uncontrolled heart rate
2. Severe sleep apnea
3. Smoking history
4. Patient’s age
5. None, aspirin is appropriate for this patient [correct response]

Record end time—

Indicate your confidence about the above answer. [11-point scale ranging from 0%-100%]

Did you know the answer beforehand? Yes / No

 

 

Scenario B.

Please answer the following question using [assigned format].

A 72-year-old female was admitted to the hospital for severe constipation. During a digital disimpaction, she developed chest pain and shortness of breath. Initial ECG revealed new ST segment depression consistent with ischemia. Initial troponin T was slightly elevated at 0.04. She was transferred to CCU. Cardiac catheterization revealed normal coronary arteries. An Echo (EF=25%; 6 months prior EF=56%) was consistent with apical ballooning syndrome.

Record start time—

*Mark only one best answer*—What is the recommendation for follow up Echo to assess ejection fraction progression?

1. 48-72 hours
2. 1-2 weeks
3. 4-6 weeks [correct response]
4. 8-10 weeks

Record end time—

Indicate your confidence about the above answer. [11-point scale ranging from 0%-100%]

Did you know the answer beforehand? Yes / No

### Participants, Group Allocation, and Procedures

We sent an email to all clinicians (practicing physicians, physicians in training, senior medical students, physician assistants, and nurse practitioners) who had used AskMayoExpert at least once (N=1474), inviting them to attend a noon study session to evaluate AskMayoExpert. There were two date options in Rochester and one at each other site. Those who were willing to participate and available at the required time came to one of the five face-to-face sessions. At each session booklets A and B were placed in a single stack with alternating format. Participants took the top booklet as they entered the room, and this determined group allocation (ie, to answer the atrial fibrillation scenario first, with the local resource, or second, using other resources). Each clinician then used a separate computer to answer the two questions using Web-based resources, as instructed. Participants were asked not to discuss the scenarios or answers with one another. No incentives were provided other than lunch during the session.

### Outcome Measures and Data Collection

Main dependent variables were accuracy of response, confidence in that response, and time to generate that response. Each scenario was associated with one multiple choice question (Textbox 2). Scenarios and questions were developed by a general internist (author FJL) and revised with input from two cardiology experts (author JL and another cardiologist). This group determined answers by reference to specific literature sources. During the session participants recorded the time they started and ended their search to answer the question. They also indicated their confidence in their answer (11-point ordinal scale ranging 0% to 100% confident) and whether they knew the answer beforehand. We asked, “What resources do you use to answer clinical questions?,” but did not verify whether they used these resources during this test session. We also collected demographic information (gender and specialty).

### Statistical Analysis

The prespecified primary outcome was the time to a correct response with at least 80% confidence. Secondary outcomes included percent correct, time to an incorrect response, and confidence in the response.

We report median rather than mean confidence score and time because these did not follow a normal distribution. To compare accuracy between resource formats across both scenarios, we used generalized linear models with a logit link function and repeated measures on subjects. To compare time and confidence between resource formats, we performed a similar repeated measures analysis using mixed effects analysis of variance on the ranked outcomes. In a sensitivity analysis, we repeated these analyses separately for practicing physicians, nonphysician practitioners, and physician trainees. The time to a confident, correct answer was evaluated with a competing risks model [15] predicted by scenario, study intervention, and the interaction of outcome type and study intervention, with repeated measures on subjects. A two-sided 5% type I error was used for all analyses. As a pilot study, we powered the study for a large effect (Cohen’s d 0.8), which required 52 participants to achieve 80% power. Author FE (a PhD statistician) planned all analyses. We used SAS 9.1.3 for all analyses.

## Results

### Participants

There were 59 clinicians that participated, including 28 practicing physicians, 14 physician assistants/nurse practitioners, 10 postgraduate physician trainees, 6 senior medical students, and 1 licensed clinical social worker. Table 1 contains additional demographic information. The 59 participants were similar to those invited, but not participating, in characteristics of gender, years of service, and training level (*P*>.10; data not shown). The proportions of participants who reported knowing the answer beforehand were similar for the local and other resources. The number of participants per session varied from 2 to 22.

### Accuracy, Confidence, and Time

Overall accuracy, confidence, and time are shown in Table 2. Answers were more often accurate when using the local resource than when using other Web-based resources, with odds ratio 6.2 (95% CI 2.6-14.5; *P*<.001) when averaged across scenarios. Time to find an answer was faster, and confidence in that answer was consistently higher, for the local resource (*P*<.001; Table 2). In a sensitivity analysis, we performed these analyses separately for practicing physicians, nonphysician practitioners, and trainees; results showed the same direction of effect, but given low power did not always reach statistical significance (data not reported).

Table 3 shows that inappropriate confidence (overconfidence) was less frequent with the local resource. Among confident clinicians (those with ≥80% confidence), the odds of being correct (vs incorrect) were 10.0 times higher for the local resource than for other resources for Scenario A (95% CI 1.4-78), and for Scenario B the odds ratio was 3.4 (95% CI 0.6-23.6).

### Time to an Accurate and Confident Response

In the primary outcome analysis, a time-to-event competing risk model, only clinicians who achieved an accurate and confident (≥80%) response were considered to have a positive outcome. In this analysis, the chance of being correct and confident at a given time was 2.5 times higher for the local resource than with other resources (95% CI 1.6-3.8; *P*<.001).

### Other Resources Used in Practice

We asked participants what resources other than AskMayoExpert they use to answer clinical questions, but did not verify that they used these resources during this test session. The resources most commonly reported were UpToDate (48/58, 83% respondents), Micromedex (38/58, 66%), PubMed and Google (34/58, 59% each), and MEDLINE (24/58, 41%).

**Table 1:** Participant demographics.

Feature		All, N=59	AskMayoExpert for Scenario A, n=30	Other for Scenario A, n=29
**Training level** ^a^ **, n (%)**				
	Staff MD	28 (47)	12 (40)	16 (55)
	PA/NP	14 (24)	10 (33)	4 (14)
	PG	10 (17)	5 (17)	5 (17)
	MS	6 (10)	3 (10)	3 (10)
	LCSW	1 (2)	0	1 (4)
**Gender** ^b^ **, n (%)**				
	Male	35 (59)	15 (50)	20 (69)
**Site** ^c^ **, n (%)**				
	Rochester, MN	23 (39)	12 (40)	11 (38)
	Jacksonville, FL	22 (37)	11 (37)	11 (38)
	Scottsdale, AZ	13 (22)	7 (23)	6 (21)
	Mankato, MN	1 (2)	0	1 (4)^c^
**Knew answer beforehand** ^d^ **, n (%)**				
	Scenario A	5 (9)	3 (10)	2 (8)
	Scenario B	11 (20)	7 (24)	4 (15)

^a^Between-groups comparison across all training levels: *P*=.38. Staff MD: staff physician; PA/NP: physician's assistant/nurse practitioner; PG: postgraduate physician trainee; MS: medical student; and LCSW: licensed clinical social worker.

^b^Between-groups comparison: *P*=.19.

^c^Between-groups comparison across all sites: *P*=1.0. One additional person participated in Mankato, but data were largely incomplete and this participant's data are not included in any analyses.

^d^Reported by participants after answering the question. Between-groups comparison, Scenario A: *P*=1.0; Scenario B: *P*=.51.

**Table 2:** Accuracy, confidence, and time to answer question.

	Scenario A			Scenario B		
	Accuracy n correct (%)	Confidence^a^ median (IQR)	Time^a^ median (IQR)	Accuracy n correct (%)	Confidence^a^ median (IQR)	Time^a^ median (IQR)
Local resource	27/30 (90)	100 (95, 100)	2 (1, 3)	24/29 (83)	90 (70, 100)	4 (3, 5)
Other Web-based resources	14/29 (48)	60 (30, 80)	3.5 (2, 8)	16/30 (53)	80 (70, 90)	4 (3, 6)

^a^Confidence measured using an ordinal scale, 0%, 10%, …100% confident; time measured in minutes; IQR: interquartile range.

**Table 3:** Accuracy of and confidence in responses.

	Incorrect, but confident^a^ n confident (%)		Correct, but not confident^a^ n confident (%)	
	Scenario A	Scenario B	Scenario A	Scenario B
Local	3/28 (11)	3/22 (14)	2/2 (100)	4/7 (57)
Other	6/11 (55)	7/20 (35)	9/18 (50)	3/10 (30)

^a^Confidence > 80%

## Discussion

### Summary of Findings

We found that accuracy was significantly higher, and overconfidence was lower, when using a concise locally developed resource (AskMayoExpert) than when using another Web-based resource selected by the participant. Time slightly favored the local resource, but the difference was not statistically significant. However, in the prespecified primary analysis, after accounting for time, the chance of correctly and confidently answering the question was 2.5 times higher for the local resource.

### Limitations and Strengths

The use of a locally developed knowledge resource, and clinical scenarios restricted to cardiology, limit the generalizability of our findings. Moreover, these scenarios could have inadvertently targeted content unique to the local resource (ie, giving it an unfair advantage), although we are not aware of such bias. We did not track the resources used when addressing the second scenario. Time was recorded by participants, and thus susceptible to error. Although we achieved overall statistical significance in the primary outcome, Scenario A accounts for the majority of the difference in time in this analysis. We had low response to our initial invitation, and although measured demographics were similar, participants could be systematically different than nonparticipants in unmeasured attributes. Confidence in the local resource could have been influenced by knowledge that local colleagues created information. Group assignment was not strictly random; but since participants used both knowledge resources during the study, neither they nor the study proctor had incentive to deliberately influence the assignment process. Moreover, the crossover design offers within-subjects control for individual differences. Another strength is the measurement of three key outcomes (accuracy, time, and confidence).

### Comparison With Prior Work

Synthesized knowledge resources (in which experts attempt to present a balanced summary of evidence, such as UpToDate, DynaMed, and MD Consult) have been compared with one another [16-19] and with unsynthesized resources (that provide access to primary literature, such as PubMed) [18,20,21] in both clinical practice and in test settings. In these studies, the synthesized knowledge resource is consistently faster and/or more accurate. The findings of the present study show a similar effect, namely, that a concise evidence-based resource designed expressly for point of care learning facilitates quick, accurate answers to clinical questions.

### Implications and Conclusions

Although this pilot study has several limitations, it demonstrates that important differences exist among knowledge resources. Specifically, a resource crafted to provide quick, concise answers at the point of care was associated with more accurate responses, and faster time to an accurate response, than other clinician-selected Web-based resources. Future research might explore how to design and implement knowledge resources more effectively, investigate how to encourage clinicians to optimally use them to enhance patient care, and determine their clinical impact on patient health and systems outcomes.

## Abbreviations

CI: confidence interval
FAQ: frequently asked questions
